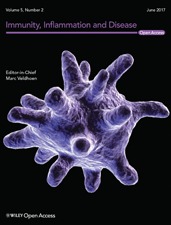# Issue Information

**DOI:** 10.1002/iid3.129

**Published:** 2017-05-04

## Abstract